# Effect of in-office bleaching gels with calcium or fluoride on color, roughness, and enamel microhardness

**DOI:** 10.4317/jced.56006

**Published:** 2020-02-01

**Authors:** Isabele Vieira, Waldemir-Francisco Vieira-Junior, Maria-Cibelle Pauli, Jéssica-Dias Theobaldo, Flávio-Henrique-Baggio Aguiar, Débora-Alves-Nunes-Leite Lima, Gislaine-Ricci Leonardi

**Affiliations:** 1 DDS student, Department of Restorative Dentistry, Piracicaba Dental School, University of Campinas, Piracicaba, São Paulo, Brazil; 2 DDS, MS, PhD, Professor, Department of Restorative Dentistry, São Leopoldo Mandic Institute and Dental Research Center, Campinas, São Paulo, Brazil; 3 MS, PhD student, Department of Translational Medicine, Federal University of São Paulo, São Paulo, SP, Brazil; 4 DDS, MS, PhD, Department of Restorative Dentistry, Piracicaba Dental School, University of Campinas, Piracicaba, São Paulo, Brazil; 5 DDS, MS, PhD, Associate Professor, Department of Restorative Dentistry, Piracicaba Dental School, University of Campinas, Piracicaba, São Paulo, Brazil; 6 MS, PhD, Professor, Faculty of Pharmaceutical Sciences, University of Campinas, Campinas, Brazil

## Abstract

**Background:**

Commercial bleaching gels with remineralizing agents were developed to reduce the adverse effects of dental bleaching. The present study evaluated the effects on teeth of in-office bleaching gels containing 35-40% hydrogen peroxide (HP) with Calcium (Ca) or Fluoride (F).

**Material and Methods:**

Bovine enamel/dentin blocks (4x4x2.5 mm) were randomly divided into the following groups (n=12): no treatment (control); 35% HP (Whiteness HP, FGM); 35% HP with Ca (Whiteness HP Blue, FGM); 40% HP with F (Opalescence Boost, Ultradent). The specimens were analyzed for color (ΔL*, Δa*, Δb*, and ΔE), roughness (Ra), and Knoop microhardness (KHN). The color and KHN data were submitted to ANOVA and Tukey’s test, while Ra values were analyzed using mixed models for repeated measures and Tukey-Kramer’s test (α=0.05).

**Results:**

The bleached groups did not exhibit statistical differences among them for color. For roughness, 35% HP provided a slight increase of Ra, which was statistically different from the control. For microhardness, 35% HP and 40% HP with F presented KHN values that were statistically lower from the control, while the 35% HP with Ca did not statistically differ from the control.

**Conclusions:**

The presence of Ca or F in bleaching gels did not interfere with bleaching efficacy. However, only the enamel exposed to the bleaching gel containing Ca obtained microhardness values similar to unbleached enamel.

** Key words:**Hydrogen peroxide, tooth bleaching, tooth bleaching agents, laboratory research.

## Introduction

High beauty standards and cosmetic care are highly valued in Dentistry. The desire to have a harmonious smile with well-aligned white teeth has become a basic standard of aesthetics, which increases the demand for dental treatments (1). The tooth color can be mediated by either extrinsic factors, such as the ingestion of foods, oral habits, and the consumption of beverages containing dyes (e.g., tea, coffee, tobacco, red wine), or intrinsic factors (e.g., hematological disorders and exposure to phenolic or iodoformic drugs) (2). Furthermore, changes to the substrate structure during dental development, such as amelogenesis, dentinogenesis imperfecta, enamel hypoplasia, and dental fluorosis; may affect the color of teeth (2).

Different methods and products for dental bleaching are available on the market. The at-home and in-office dental bleaching methods are considered effective and relatively safe when supervised by a dentist (3,4). In the at-home bleaching therapy, the professional indicates a low-concentration bleaching agent used in a high-frequency regime; alternatively, in-office dental bleaching occurs using high-concentration agents that are applied to teeth (5). Another controversial alternative is over-the-counter whitening products, such as dentifrices, strips, and mouthwashes; which contain whitening agents and are sold directly to the consumer (5).

The bleaching agent most commonly used in in-office treatments is 30-40% hydrogen peroxide (HP), which is effectively associated with chemical or physical catalysts (6). The mechanism of dental bleaching is dynamic, complex, and based on HP penetration/diffusion into the enamel and dentin to interact from the HP react with the stain molecules and converts them into smaller molecules, altering the optical properties of the dental substrate (5,7). The reaction products are lower in molecular weight when compared to the original stain molecule, and these properties make the products easier to remove from the dental structure (7).

Regardless of the technique and bleaching agent employed, adverse effects have been reported, to include: tooth sensitivity (8); changes in surface morphology; and changes to the physical-chemical properties of the dental hard tissues such as an increase in enamel roughness, a decrease in surface microhardness, and an alteration in mineral content (9-13). Some remineralizing compounds, such as fluoride, calcium, bioactive glass, arginine/calcium carbonate, and nanohydroxyapatite; have been investigated to minimize these adverse effects (10,12,14-16). These compounds could be used before or after treatment, or incorporated into gels to prevent demineralization or enhance remineralization during the bleaching therapy.

Commercial bleaching gels that contain calcium or fluoride were developed to control adverse effects. However, in order to become a widely used approach, it is necessary to evaluate the effects of the remineralizing agents incorporated into bleaching gels, including the validation of bleaching efficacy or the avoidance of undesirable effects, which is associated with an alteration in the physical properties of teeth. The aim of this *in vitro* study was to evaluate commercially available in-office bleaching gels based on 35-40% HP, without or with calcium or fluoride, on the bleaching efficacy, roughness, and microhardness of enamel. The null hypotheses tested were: 1) the presence of calcium or fluoride in bleaching gels would not affect the bleaching efficacy; 2) in-office dental bleaching would not alter the roughness or surface microhardness of enamel; and 3) the presence of calcium or fluoride in bleaching gels would not protect the enamel against the potential changes in roughness or surface microhardness.

## Material and Methods

-Preparation of specimens

Bovine incisors were extracted and stored in a 0.01% thymol solution at 4°C for up to 30 days until use. Blocks with dimensions of 4 x 4 x 2.5 mm, with 1 mm of enamel and 1.5 mm of dentin, were obtained from the middle third of the buccal surface, using a slow-speed, water-cooled diamond saw (Isomet - Buehler Ltd, Lake Bluff, IL, USA). The specimens were serially planed using 600-, 1000-, and 2000-grit SiC papers (Buehler Ltd) and polished with felts (TOP, RAM, and SUPRA - Arotec, Cotia, SP, Brazil) and metallographic diamond pastes. All specimens were placed in an ultrasonic machine containing distilled water for 10 min (Marconi, Piracicaba, SP, Brazil) between the polishing steps and at the end of the polishing procedures in order to remove residual particles. The specimen surfaces, with the exception of the enamel surface, were protected using an acid-resistant varnish (Risqué, Barueri, SP, Brazil). Before (24 h prior) and during the experiment, all prepared specimens were stored in a remineralizing solution (artificial saliva) containing 1.5 mM Ca, 0.9 mM P, 150 mM KCl, 0.05 µg F/mL, and 0.1 M Tris buffer at pH 7.0 (17), which was renewed every.

-Specimen Allocation, group division, and bleaching treatments

Forty-eight specimens were allocated into four groups (n=12). The initial L* value of each specimen was used to stratify and allocate specimens. The L* represents a significant color coordinate, the L* stratification reduced the initial variability among the groups, enabling adequate statistical comparisons. The acquisition of the L* coordinate is described in the Color Measurements section, which follows.

The groups were submitted to different bleaching treatments and the application technique for each treatment was performed following the manufacturer’s instructions. The information regarding the products used in the present study is presented in  Table 1. The study design was based on the following groups:

**Table 1:**
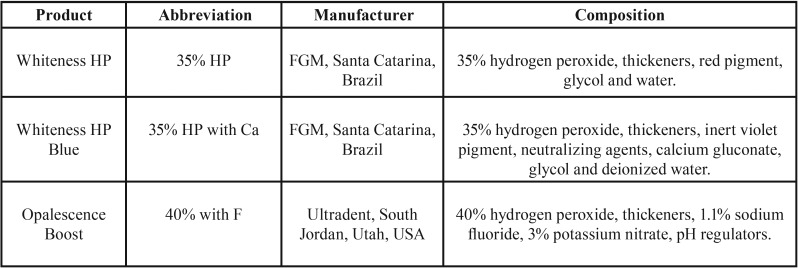
Products used in the present study, according to the manufacturer.

Control: unbleached enamel, no treatment;

35% HP: treatment with 35% hydrogen peroxide (Whiteness HP, FGM, SC, Brazil). Two sessions were performed with an interval of 7 days. In each session, the gel was applied three times, each for a duration of 15 min (45 min total).

35% HP with Ca: treatment with gel containing 35% hydrogen peroxide and calcium (Whiteness HP Blue, FGM, SC, Brazil). Two sessions were performed with an interval of 7 days. In each session, the gel was applied once for 40 min.

40% HP with F: Treatment with gel containing 40% hydrogen peroxide and fluoride (Opalescence Boost, Ultradent, UT, USA). Two sessions were performed with an interval of 7 days. At each session, the gel was applied twice, each for a duration of 20 min (40 min total).

After the treatment sessions, the specimens were washed with distilled water, dried with absorbent paper, and stored in new remineralizing solution.

-Color Measurements

For color analyses, the specimens were evaluated inside a light chamber (GTI, Newburg, NY, USA) to standardize the environment. The color was analyzed using a reflectance spectrophotometer (CM 700d, Minolta, Osaka, Japan) that had been previously calibrated. The values were quantified using the CIE L*a*b* system. The L* coordinate presents the luminosity (white-black axis), the a* coordinate presents the green-red axis, and the b* coordinate presents the blue-yellow axis. The readings were completed before and after the treatments. The results were expressed by a Δ coordinate, representing the difference between the final and baseline coordinate values. The general color change was calculated using the following equation: ΔE = [(ΔL*)2+(Δa*)2+(Δb*)2]1/2.

-Surface roughness analysis

Three readings per specimen were performed in different directions from points equidistant on the enamel surface using a profilometer tester (Surfcorder SE 1700, Kosaka, Tokyo, Japan). The profilometer tester was standardized with a cutoff of 0.25 mm, a reading length of 1.25 mm, and a velocity of 0.05 mm/s. The average of three readings was determined using the arithmetic roughness (Ra) parameter. The surface roughness was evaluated before (baseline) and 24 h after the final bleaching session.

-Surface microhardness analysis

Enamel microhardness was evaluated using a hardness tester (HMV-2000, Shimadzu, Tokyo, Japan), with indentations made using a Knoop indenter (5 g/10 s). Five indentations were made for specimen with a distance of 100 µm between them. The average of these indentations was calculated to express the Knoop hardness number (KHN) for each specimen, which was considered the final enamel microhardness. The KHN evaluation was performed at the end of the experiment because the microhardness analysis marks the surface, which could interfere with the roughness analysis.

-Statistical analysis

The color variables (ΔL*, Δa*, Δb*, and ΔE) and microhardness values (KHN) were evaluated using one-way analysis of variance (ANOVA) and Tukey’s test. The roughness results (Ra) were analyzed using mixed models for repeated measures and Tukey-Kramer’s test using the SAS software (SAS® Studio 3.5: User’s Guide. Cary, NC, USA). For all analyses, the significance level was set at 0.05.

## Results

The color results are presented in  Table 2. The bleached groups (35% HP, 35% HP with Ca and 40% HP with F) did not differ statistically from each other for ΔL* (*p* = 0.66), Δa* (*p* = 0.65), Δb* (*p* = 0.35), and ΔE (*p* = 0.69). For ΔL*, the bleached groups statistically differed from the unbleached control group (*p* < 0.01), demonstrating a positive variation along the L* axis. For Δa*, all bleached groups statistically differed from the control group (*p* < 0.01), with a decrease in a* values. For Δb*, the bleached groups statistically differed from the unbleached control group, with negative values for the b* coordinate (*p* < 0.01). Regarding the general color variation of the specimens (ΔE), the bleached groups presented statistical differences from the control group (*p* < 0.001).

**Table 2:**
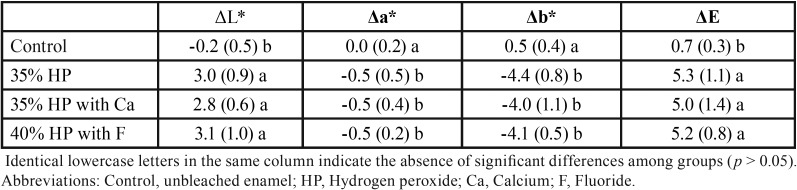
Mean (SD) for ΔL*, Δa*, Δb*, and ΔE, based on bleaching procedures.

**Figure 1:**
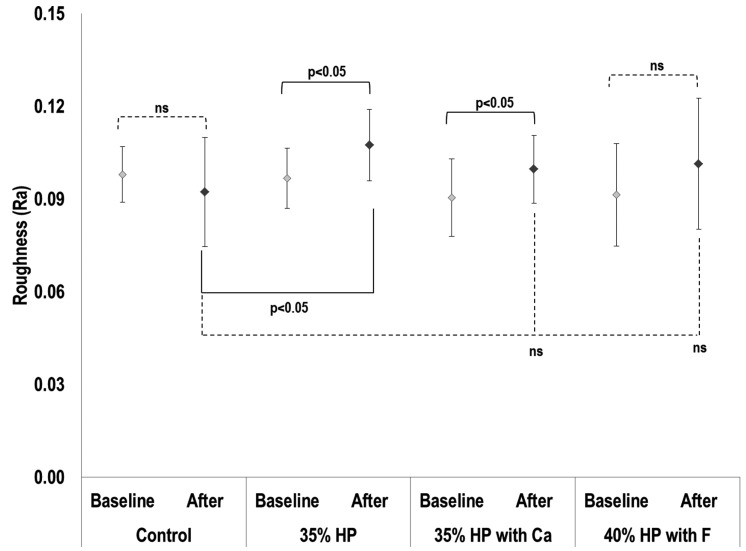
Roughness values for baseline and final values, based on treatments. Legend: *p*<0.05, significant statistical differences between groups (continuous line). Ns, no significant difference between groups (dotted line). Statistical analysis: bleaching agent (*p* = 0.13), time (baseline vs. after, *p* = 0.0003), interaction (*p* = 0.0005). Abbreviations: Control, unbleached enamel; HP, Hydrogen peroxide; Ca, Calcium; F, Fluoride.

**Figure 2:**
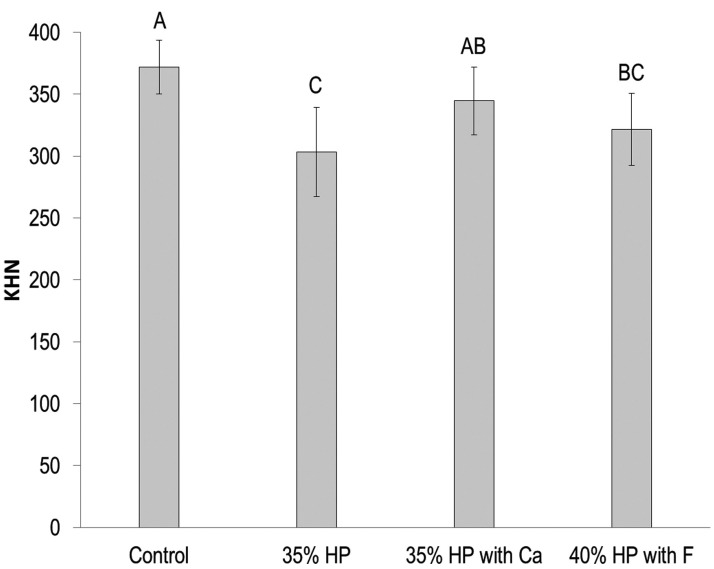
Enamel microhardness (KHN) based on the bleaching procedures. Identical uppercase letters (above the error bar) indicate the absence of significant differences among groups (*P* > 0.05). Abbreviations: Control, unbleached enamel; HP, Hydrogen peroxide; Ca, Calcium; F, Fluoride.

The analysis of roughness (Ra) is presented in Figure 1. The statistical analysis showed an absence of effect for the bleaching agent used (*p* = 0.1354), a significant effect for time (*p* = 0.0003), and an interaction of both factors (bleaching agent*time, *p* = 0.0005). Based on Figure 1, there was no significant difference between the groups for baseline values (*p* > 0.05). There was a significant increase in the roughness values for 35% HP and 35% HP with Ca (*p* = 0.0003), which presents the comparison between baseline and final values. Considering the final values of Ra, the 35% HP group presented Ra values significantly higher than the control group (*p* < 0.05). Based on the enamel microhardness results (Fig. 2), 35% HP and 40% HP with F presented the smallest KHN values, which were statistically different from the control group (*p* < 0.01) and statistically similar to each other (*p* = 0.21). The 35% HP with Ca did not statistically differ from the control group (*p* > 0.05); although it was statistically different from the 35% HP group (*p* < 0.05).

## Discussion

Dental bleaching is an effective and relatively safe treatment (3,5,8). Nevertheless, dental bleaching can cause adverse effects on tooth structures (8), such as microhardness changes and mineral content alterations, according to the study design (18). New formulations for bleaching gels containing Ca or F that are on the market are intended to help minimize these deleterious effects. The results of this study indicate that the incorporation of F or Ca did not interfere with the bleaching efficacy of the treatment because the color analyses demonstrated an increase of L* value (black-white axis), a decrease of b* values (blue-yellow axis), and the means of general color change (ΔE) were greater than 4.2 units, which is considered a standard value of clinical acceptance of color difference (19). Thus, null hypothesis 1 was accepted because the presence of Ca or F in the bleaching gels did not interfere with the bleaching efficacy of the treatment.

In the present study, the specimens were not initially pigmented by coffee or red wine because that might have created topographic alteration due to the acidic pH of these solutions, which could interfere with the roughness analysis. The standardization of two application sessions for all bleaching agents was considered sufficient to promote clinically relevant color changes, as demonstrated by the ΔL*, Δb* and ΔE values. Moreover, the study model allowed the physical properties of enamel to be evaluated and the effect of remineralizing agents incorporated in the gel.

Previous studies (20,21) have suggested that the addition of F in the bleaching gel promotes a reduction in enamel demineralization during bleaching therapies. However, based on the present results, the presence of F did not seem to provide additional benefits to enamel microhardness. The effect of F could be minimized by the higher HP concentration of the commercial gel used in the present study (40%), as there is a proportional relationship between mineral loss and concentration of bleaching agent (22). The alterations in the enamel properties described in the present study for 35% HP or 40% HP with F can be explained by the oxidizing effect promoted by HP (7) or the acidic pH of the bleaching agents that stimulate the demineralization of the dental structure (23). Thus, based on the current results, null hypothesis 2 was rejected because the in-office dental bleaching with 35% HP negatively affected the roughness and microhardness of the enamel.

On other hand, the group exposed to the bleaching gel containing 35% HP with Ca presented similar KHN values to those found in the unbleached enamel. The presence of Ca in the HP gel could promote protection of the enamel against mineral loss; however the interaction mechanism of Ca during demineralizing events is not fully understood. Ca could act during demineralization events by diffusion through the dental structure (21) or surface precipitation that physically acts against the demineralizing action of the HP gel (24). Considering this finding, null hypothesis 3 was partially rejected because a positive effect in protecting against a decrease in microhardness values was found for the bleaching gel containing 35% HP with Ca.

In this study, an increase in roughness occurred; however, although the roughness of the enamel could be associated with mineral loss, topography alteration, or modification of light reflectance (25); the values were considered relatively small when compared to the unbleached control group and were considered similar to the results previously reported (10). This roughness variation possibly is clinically irrelevant, although it is necessary to study the clinical bleaching effects on the tooth structure. The alteration in roughness can be restored by human saliva or over a longer time of storage in artificial saliva (13), in which ions are available to remineralize or prevent demineralization of dental structures during erosive/demineralizing events, including dental bleaching (18). Although this slight roughness increase (approximately 0.01 in Ra values) was detected in the 35% HP and 35% HP with Ca groups, only 35% HP differed from the final values of unbleached control. Therefore, when considering the final values of Ra, the groups exposed to the commercial bleaching gel containing F behaved as the intact unbleached enamel, whereas the roughness variation was not detected in the 40% HP with F group.

Previous studies (14,15) indicated that the addition of Ca and F to the bleaching gels did not provide additional protection for the enamel. These findings partially contrast with the results obtained in the present study, where the 35% HP with Ca group showed microhardness values similar to the unbleached control group. Nevertheless, the commercial bleaching agents investigated in this study are available in different HP concentrations, application modes, and total exposure times, which make direct comparisons difficult. Thus, a total inactivity of fluoride in preventing the demineralization events commonly associated with dental bleaching cannot be determined, whereas it was not possible to investigate a bleaching gel containing F and 35% HP simultaneously. With the growing demand for dental bleaching and the development of new formulations, the products with F, Ca, or other agents can absolutely be improved to combine the benefits that these ions provide to the enamel in dental aesthetic treatments. However, new materials must be designed to provide more conservative and effective procedures based on validation by *in vitro*, in situ, and *in vivo* studies.

## Conclusions

Commercial bleaching gels containing remineralizing agents, such as calcium or fluoride, promote the same bleaching efficacy as conventional bleaching gels. However, when considering the formulations investigated in the present study, the incorporation of calcium into a bleaching gel minimized negative effects to microhardness.
